# Discrimination between NSIP- and IPF-Derived Fibroblasts Based on Multi-Parameter Characterization of Their Growth, Morphology and Physic-Chemical Properties

**DOI:** 10.3390/ijms23042162

**Published:** 2022-02-15

**Authors:** Barbara Orzechowska, Kamil Awsiuk, Dawid Wnuk, Joanna Pabijan, Tomasz Stachura, Jerzy Soja, Krzysztof Sładek, Joanna Raczkowska

**Affiliations:** 1 Institute of Nuclear Physics Polish Academy of Sciences, Radzikowskiego 152, 31-342 Krakow, Poland; barbara.orzechowska@ifj.edu.pl (B.O.); Joanna.Pabijan@ifj.edu.pl (J.P.); 2 The Marian Smoluchowski Institute of Physics, Jagiellonian University, Łojasiewicza 11, 30-428 Krakow, Poland; kamil.awsiuk@uj.edu.pl; 3 Jagiellonian Center of Biomedical Imaging, Jagiellonian University, Łojasiewicza 11, 30-348 Krakow, Poland; 4 Department of Cell Biology, Faculty of Biochemistry, Biophysics and Biotechnology, Jagiellonian University, Gronostajowa 7, 30-387 Krakow, Poland; dawid.wnuk@uj.edu.pl; 5 2nd Department of Internal Medicine, Jagiellonian University Medical College, Jakubowskiego 2, 30-688 Krakow, Poland; tomasz.stachura@interia.pl (T.S.); jerzysoja@op.pl (J.S.); mmsladek@cyfronet.pl (K.S.)

**Keywords:** interstitial lung diseases (ILD), idiopathic pulmonary fibrosis (IPF), nonspecific interstitial pneumonia (NSIP), fibroblasts, substrate elasticity, force spectroscopy, ToF-SIMS

## Abstract

Background: The aim of the research presented here was to find a set of parameters enabling discrimination between three types of fibroblasts, i.e., healthy ones and those derived from two disorders mimicking each other: idiopathic pulmonary fibrosis (IPF), and nonspecific interstitial pneumonia (NSIP). Methods: The morphology and growth of cells were traced using fluorescence microscopy and analyzed quantitatively using cell proliferation and substrate cytotoxicity indices. The viability of cells was recorded using MTS assays, and their stiffness was examined using atomic force microscopy (AFM) working in force spectroscopy (FS) mode. To enhance any possible difference in the examined parameters, experiments were performed with cells cultured on substrates of different elasticities. Moreover, the chemical composition of cells was determined using time-of-flight secondary ion mass spectrometry (ToF-SIMS), combined with sophisticated analytical tools, i.e., Multivariate Curve Resolution (MCR) and Principal Component Analysis (PCA). Results: The obtained results demonstrate that discrimination between cell lines derived from healthy and diseased patients is possible based on the analysis of the growth of cells, as well as their physical and chemical properties. In turn, the comparative analysis of the cellular response to altered stiffness of the substrates enables the identification of each cell line, including distinguishing between IPF- and NSIP-derived fibroblasts.

## 1. Introduction

Idiopathic interstitial pneumonias (IIP) belongs to the broad and heterogeneous spectrum of pulmonary disorders classified as interstitial lung diseases (ILD), related to the inflammatory process affecting the interstitium of the lungs, frequently accompanied by fibrosis [1,2]. IIPs represent a group of diseases of unknown etiology, unpredictable evolution, and limited treatment options [3,4]. The impact of various factors on the origin and progression of ILD diseases, as well as on the proper diagnosis and prognosis of patients, have been intensively studied, including research on the impact of connective tissue diseases, diseases with granuloma formation, organic dust, certain drugs, genetic factors, and the composition of the microbiome in lung tissues [5,6,7]. The most frequent forms of IIP are idiopathic pulmonary fibrosis (IPF), and nonspecific interstitial pneumonia (NSIP) [8]. Both diseases have similar clinical presentations and, in the past, were even considered to be one entity [9,10]. Although the symptoms of IPF and NSIP, including a long-term cough and dyspnea [11], are mostly identical, there are significant differences in their course and management [9,12,13]. IPF occurs predominantly in males and has a very poor prognosis [9,13,14], with the median survival of only 3–5 years after diagnosis [15,16]. A US study reported the prevalence of IPF to be 10–42/100,000 in men and 4–17/100,000 in women, and the number of new IPF cases is projected to increase in the coming years, doubling by 2030 [17]. European studies suggest that, in the EU, up to 40,000 new patients will be diagnosed with IPF each year [18]. The incidence of IPF is rising with age, and its prevalence in the population older than 75 years of age is increasing, reaching more than 200/100,000 in Italy [19]. On the other hand, patients with NSIP are on average 10 years younger, and are more commonly women [20,21]. The 5-year mortality rate for NSIP patients is less than 20% [13,22,23]. Moreover, patients with NSIP usually have a good reaction to steroid treatment, whereas patients with IPF do not. Although currently no curative treatment for IPF has been developed, antifibrotic drugs (pirfenidone and nintedanib) have been shown to slow the progression of lung fibrosis in IPF patients, but are associated with significant side effects [24,25,26,27,28].

As clinical, radiological, and pathological findings of IPF and NSIP may be very similar, reaching the proper diagnosis is difficult. Despite the ‘gold standard’ for IPF diagnosis proposed in 2018 by a multidisciplinary committee of IPF experts [29,30,31], there are no good markers of the disease. The proposed criteria are often arbitrary, and the diagnostic process is frequently time consuming. In both IPF and NSIP, the diagnosis is based on the exclusion of all alternative disorders [9,21]. Differential diagnosis can be very challenging, especially considering that ILDs occur frequently not as a separate disorder, but as part of a manifestation of connective tissue diseases (e.g., as a relevant extra-articular manifestation of rheumatoid arthritis) [32,33]. Therefore, the diagnostic process is based on the integrated findings of specialists in pulmonology, radiology, and pathology, and the results of sophisticated examination techniques, such as high-resolution computed tomography (HRCT), thin-section CT (TSCT), and in some cases lung biopsy [12,33,34,35,36]. These requirements make the diagnostic process long, expensive, and complicated, and mean that new, reliable, and fast diagnostic methods are highly required [29,30,31].

In addition to the urgent need for diagnostic tools, the issues concerning the pathophysiology behind both disorders are still not fully understood. The precise mechanisms leading to the development of NSIP are unclear, but chronic inflammation of lung tissue seems characteristic of this disorder [37]. The driving mechanisms of IPF are also unknown. Until recently, the occurrence of IPF was associated with uncontrolled inflammation, as for NSIP [38]. However, nowadays, it seems that the pathomechanism of this disease is associated rather with repeated episodes of alveolar epithelial cell injuries, followed by dysfunctional healing mechanisms [38,39,40], and excessive production and accumulation of extracellular matrix (ECM) molecules, such as collagens [41,42]. This effect, leading to an increase in tissue stiffness, may be considered the crucial issue responsible for fibrogenesis [43], because the differentiation of myofibroblasts may be altered by the elasticity of the substrate [44]. Although the impact of mechanical properties of the substrate on the function of IPF fibroblasts has already been examined [45,46,47,48], no analogous research for NSIP-derived cells has been performed. Additionally, disorders of the biosynthesis and metabolism of cholesterol [49], as well as the abundance of various ECM components [50,51], postulated for IPF, may strongly affect the mechanical properties of cells. Increasing the stiffness of single fibroblasts may influence the mechanically-derived signals transmitted through the ECM, affecting the lungs at the tissue level [52,53]. Due to changes in cytoskeletal organization, IPF and NSIP-derived fibroblasts should differ in their mechanical properties [54]. Therefore, the interactions between these cells and substrates of different elasticities may be an extremely important factor in the cellular response, which needs detailed examination to establish the role of these interactions in the development of IPF and NSIP lung impairments [54].

On the other hand, chronic inflammation of the lung tissue and fibrosis also affect the biochemical properties of the tissue, and the subtle balance of compounds important for normal lung physiology, such as the cholesterol and lipoproteins present in pulmonary surfactant [55,56,57], as well as the ECM constituents [58]. Significant changes in the abundance of IPF lung matrisome components, such as different types of collagen, glycoproteins, proteoglycans, and ECM regulators as compared to normal tissue were reported by I. Germanguz et al. [50]. In turn, research performed by E. R. Parra et al. [51], points to the important role of the collagen/elastic system in vascular remodeling, which differs according to the adaptive responses to injury that occur in NSIP and IPF. Additionally, calcification in a collagen matrix, known as diffuse pulmonary ossification (DPO), has been observed in patients with fibrosing ILD [59,60]. A significantly higher prevalence of DPO was observed in patients with IPF, compared to those with NSIP [59]. Furthermore, the role of calcium-activated potassium channels on the activation and transdifferentiation of fibroblasts into myofibroblasts, potentially involved in the fibrotic process, was recently recognized [61,62,63,64]. Therefore, differences in the chemical structure of NSIP- and IPF-derived fibroblasts may also potentially serve as a parameter enabling unambiguous discrimination between them.

In this study, we examined the chemical composition, and the impact of substrate elasticity, on the properties of three cell lines derived from IPF and NSIP, as well as healthy cells, in order to find a set of parameters enabling unambiguous discrimination between the cell lines. The growth, morphology, and viability of cells were traced using fluorescence microscopy and colorimetric MTS assays, respectively. The stiffness of cells was examined using atomic force microscopy (AFM) working in force spectroscopy (FS) mode. In turn, two types of polydimethylsiloxane (PDMS) substrates with elasticities equal to 600 kPa (soft PDMS) and 1.5 MPa (stiff PDMS), as well as glass (E = 72 GPa) as control sample, were used to determine the impact of substrate elasticity on the properties of cells. Finally, the chemical composition of the cells was determined using time-of-flight secondary ion mass spectrometry (ToF-SIMS) combined with Multivariate Curve Resolution (MCR) and Principal Component Analysis (PCA), which comprise a powerful set of analytical tools providing information about very subtle differences between examined samples. The research performed shows that unambiguous discrimination between IPF and NSIP is possible at a cellular level, due to the different physical and chemical properties of IPF- and NSIP-derived fibroblasts. However, the distinction is significantly more evident when the cellular response to the altered stiffness of the substrate is considered, especially for stiffer PDMS substrates. Therefore, we believe that the mechanical properties of the cells may serve as prospective diagnostic biomarkers, enabling fast and reliable identification of IPF- and NSIP-derived fibroblasts, and might be seriously considered as an important factor when constructing novel diagnostic tools dedicated for clinicians.

## 2. Results

### 2.1. Cell Growth

#### 2.1.1. Fluorescence Microscopy

**Figure 1:**
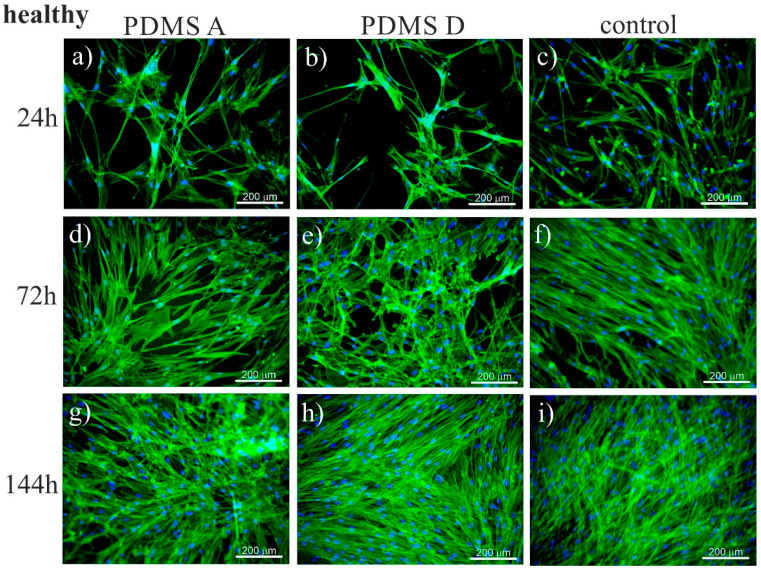
Fluorescence micrographs of the actin cytoskeleton (green) and nuclei (blue), recorded for healthy fibroblasts cultured for 24 (**a**–**c**), 72 (**d**–**f**), and 144 h (**g**–**i**) on PDMS A (**a**,**d**,**g**), PDMS D (**b**,**e**,**h**), and control samples (glass, (**c**,**f**,**i**)).

**Figure 2:**
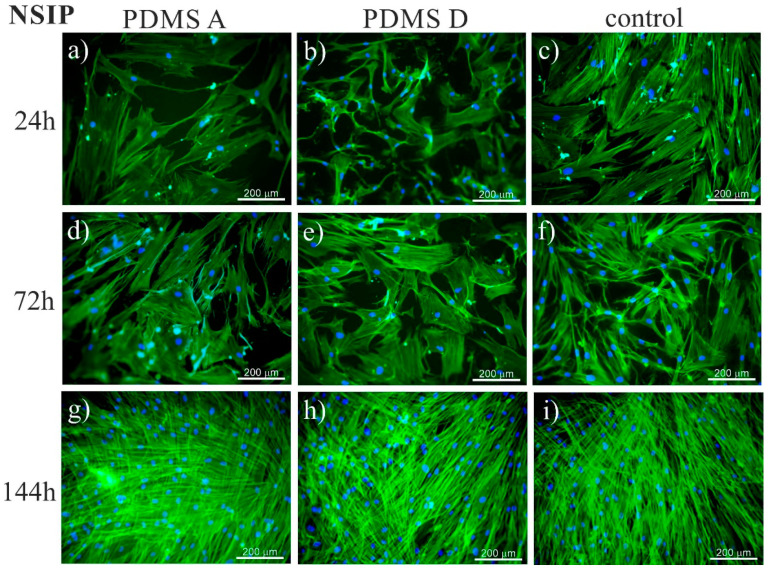
Fluorescence micrographs of the actin cytoskeleton (green) and nuclei (blue), recorded for NSIP-derived fibroblasts cultured for 24 (**a**–**c**), 72 (**d**–**f**), and 144 h (**g**–**i**) on PDMS A (**a**,**d**,**g**), PDMS D (**b**,**e**,**h**), and control samples (glass, (**c**,**f**,**i**)).

**Figure 3:**
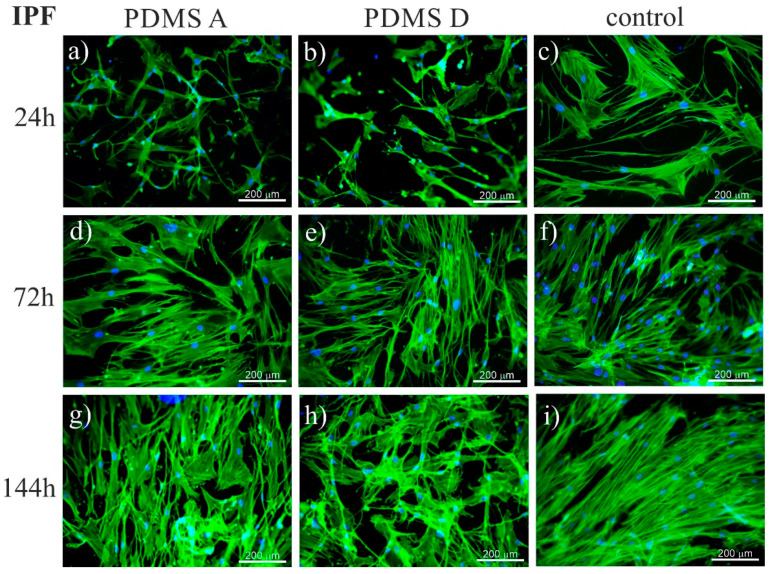
Fluorescence micrographs of the actin cytoskeleton (green) and nuclei (blue), recorded for IPF-derived fibroblasts cultured for 24 (**a**–**c**), 72 (**d**–**f**), and 144 h (**g**–**i**) on PDMS A (**a**,**d**,**g**), PDMS D (**b**,**e**,**h**), and control samples (glass, (**c**,**f**,**i**)).

To trace the impact of substrate elasticity on the growth of fibroblasts derived from NSIP and IPF, as well as healthy cells, they were cultured for 24 h, 72 h, and 144 h on soft (PDMS A), stiff (PDMS D) and control (glass) substrates. Then, they were fixed and fluorescently stained to visualize the actin cytoskeleton (green) and nuclei (blue). Representative fluorescence micrographs are presented in Figure 1 (healthy cell line), Figure 2 (NSIP-derived cell line), and Figure 3 (IPF-derived cell line).

**Figure 4:**
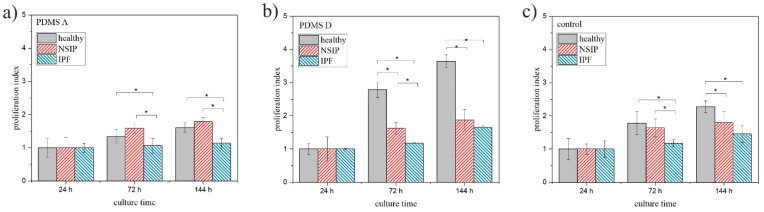
Proliferation index determined for healthy (gray), NSIP- (red), and IPF-derived (blue) fibroblasts cultured on PMDS A (**a**), PDMS D (**b**), and control (glass, (**c**)). Statistical significance was achieved for *p* < 0.01 (*).

**Figure 5:**
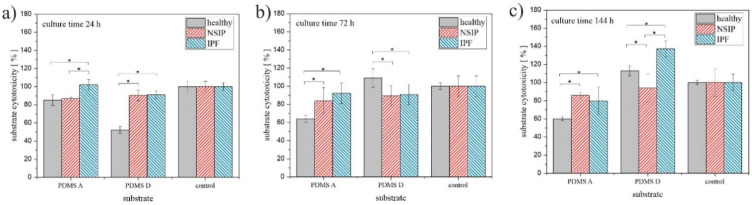
Substrate cytotoxicity determined for healthy (gray), NSIP- (red), and IPF-derived (blue) fibroblasts cultured for 24 h (**a**), 72 h (**b**), and 144 h (**c**). Statistical significance was achieved for *p* < 0.01 (*).

To analyze the impact of substrate elasticity on the growth of cells in a quantitative way, two parameters were calculated for each cell line and substrate used in this study—proliferation index, defined as the ratio between the number of cells after a given culture time and their amount after 24 h of culturing (Figure 4), and substrate cytotoxicity, defined as the ratio between the number of cells cultured on PDMS A or D, and the control substrate (Figure 5). Cells were counted using software developed in our laboratory [65]. Statistical significance was achieved for *p* < 0.01 (marked with *).

Another parameter characterizing the growth of cells on different substrates, i.e., substrate cytotoxicity, is presented in Figure 5.

#### 2.1.2. MTS

**Figure 6:**
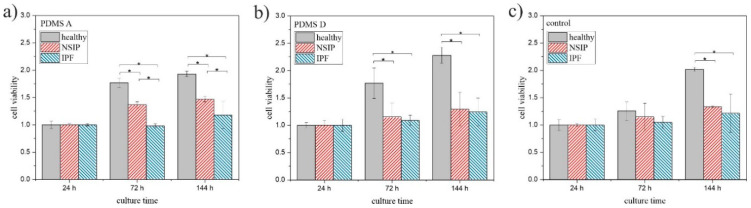
MTS values determined for healthy (gray), NSIP- (red), and IPF-derived (blue) fibroblasts cultured on PMDS A (**a**), PDMS D (**b**), and control sample (glass, (**c**)). Statistical significance was achieved for *p* < 0.01 (*).

To validate the results obtained using fluorescence microscopy, the viability of the cells was examined independently using MTS colorimetric tests (Figure 6). For all cell lines, the number of viable cells, normalized by their amount after 24 h of incubation, grows with culture time, but the growth rate depends strongly on the cell type. Statistical significance was achieved for *p* < 0.01.

### 2.2. Elasticity

The elasticity of fibroblasts was measured using AFM-based force spectroscopy. The measurements were performed on substrates of different stiffness (PDMS A—600 kPa, PDMS D—1.5 MPa, glass—72 GPa), for three indentation depths, equal to 200, 400, and 600 nm, and providing information from different cell areas [66,67,68]. The resulting Young moduli values are presented in Table 1.

**Figure 7:**
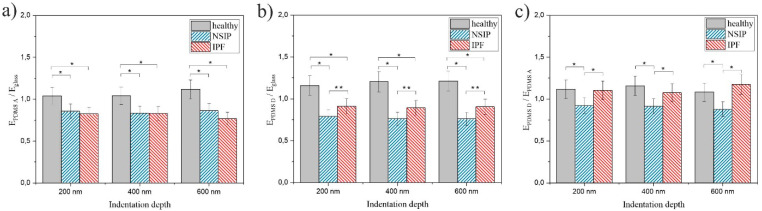
The ratio between Young moduli E_PDMS A_/E_glass_ (**a**), E_PDMS D_/E_glass_ (**b**), and E_PDMS D_/E_PDMS A_ (**c**) calculated for healthy (gray), NSIP- (blue), and IPF-derived (red) fibroblasts cultured on PMDS A, PDMS D, and glass (control). Statistical significance was achieved for *p* < 0.01 (*) or *p* < 0.05 (**).

Careful analysis of the results presented in Table 1 provides another possibility for discriminating between cells, based on the comparison of their stiffness on different substrates, expressed as a ratio between Young moduli. Ratios were calculated for PDMS A and the control sample (Figure 7a), PDMS D and the control sample (Figure 7b), and both PDMS substrates (Figure 7c). Statistical significance was achieved for *p* < 0.01 (marked as *) or *p* < 0.05 (marked as **).

### 2.3. Chemical Analysis

**Figure 8:**
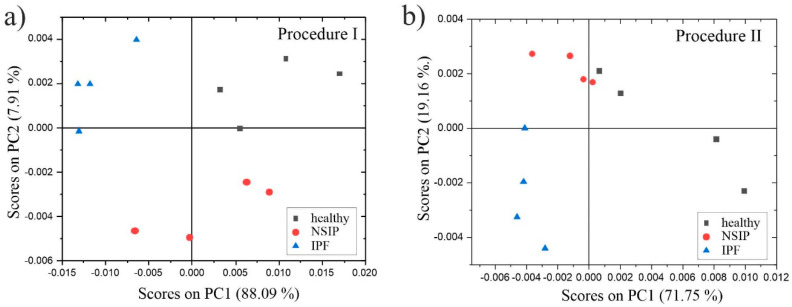
PCA score plots determined for healthy (black squares), NSIP (red circles), and IPF-derived (blue triangles) fibroblasts fixed to the surface following Procedure I (**a**), and Procedure II (**b**).

The determine the chemical composition of the studied fibroblasts, ToF-SIMS measurements were performed, accompanied by MCR and PCA analyses, which provide information about any subtle differences in chemical composition. To obtain information not only for the cell surface, but also from the deeper regions, pre-spattering was applied prior to the measurements. ToF-SIMS mass spectra were recorded for each cell line separately for both preparation methods. Since preparation methods strongly impact cell surfaces, two different peak sets were chosen, containing 96 and 125 ToF-SIMS signals, for Procedure I and Procedure II, respectively. Next, the collected data sets were analyzed by principal component analysis (PCA) to find the differences in surface chemistry between different cell lines. Figure 8 presents the PCA score plots for cells fixed to the surface following Procedure I (Figure 8a), and Procedure II (Figure 8b). For both data sets, the PCA outcome shows the separation between the studied groups of cell lines for the second principal component (PC2) plotted versus the first principal component (PC1).

Moreover, PC1, which captures the largest variance percentage, distinguished between two reference cell lines, i.e., healthy (black squares) and IPF-derived (blue triangles) fibroblasts, in both cases. Analysis of the corresponding loadings plots enables the finding of specific masses that dominate the differentiation between the studied cell types (Table 2). To identify the masses that contribute the most to the data separation, we followed the procedure described in our earlier publications [69,70]. In this procedure, the threshold values were determined based on the standard deviations (SD) of the PC components, calculated using the loadings plots.

For Procedure II, the healthy fibroblasts had positive scores, whereas both disordered lines had negative scores for PC1. This indicates that masses with loadings for PC1 higher than 1SD_PC1_ were the most characteristic for healthy cell lines (Table 3). Moreover, second principal component analysis separated NSIP-derived cells with positive scores, and IPF-derived fibroblasts with negative scores for PC2. The simultaneous analysis of loadings for PC1 and PC2, under conditions that: (1) PC1 loading values are greater than 0, and (2) PC2 loading values are lower than −1SD_PC2_ or greater than 1SD_PC2_, allow the identification of masses characteristic of IPF- and NSIP-derived fibroblasts, respectively. Table 3 presents a summary of loadings plot analyses that reveals the ToF-SIMS signals that are the most characteristic of each cell line.

Additionally, for cell lines prepared following Procedure II, a multivariate curve resolution (MCR) analysis of the ToF-SIMS cell images was performed. The goal of MCR analysis is to find a set of “pure” components that describe the differences within the ToF-SIMS images [71,72]. MCR provides component (factor) images where the brighter regions show a higher intensity of the signals present in the corresponding factor spectra (loading), whereas darker regions show areas of lower or no intensity for those peaks.

**Figure 9:**
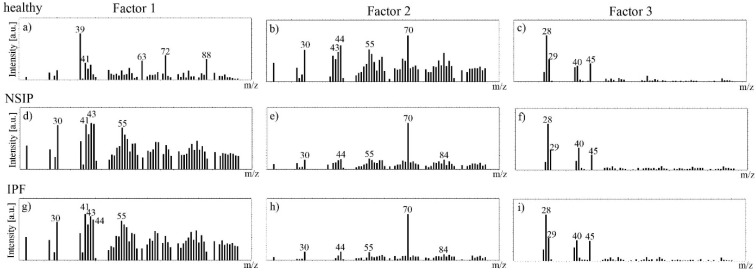
MCR loadings of Factor 1 (**a**,**d**,**g**), 2 (**b**,**e**,**h**), and 3 (**c**,**f**,**i**) spectra determined for healthy (**a**–**c**), NSIP- (**d**–**f**), and IPF-derived (**g**–**i**) fibroblasts.

Three MCR factors were calculated for the ToF-SIMS data recorded for each cell line analyzed (Figure 9). A brief look at the corresponding loadings demonstrates that the peaks in the MCR Factor 3 spectra are mainly attributable to the silicon substrate: 28 (Si^+^), 29 (SiH^+^), 45 (SiOH). In turn, Factors 1 and 2 clearly present differences in chemistry between the healthy and both disordered cell types. The MCR spectra for Factors 1 and 2 calculated for NSIP- and IPF-derived cells are similar, and refer to the same “pure” components. In turn, the MCR spectrum for Factor 1 obtained for healthy cells is completely different, which points to an additional component in cell chemistry in this case. The main peaks in the MCR spectra for Factor 1 calculated for healthy fibroblasts refer to the following ToF-SIMS signals: 39 (K^+^), 41 (^41^K), 63 (C_2_ONa^+^), 72 (CNNa_2_^+^), 88 (CNONa_2_^+^), and are in agreement with the results of the PCA analysis.

**Figure 10:**
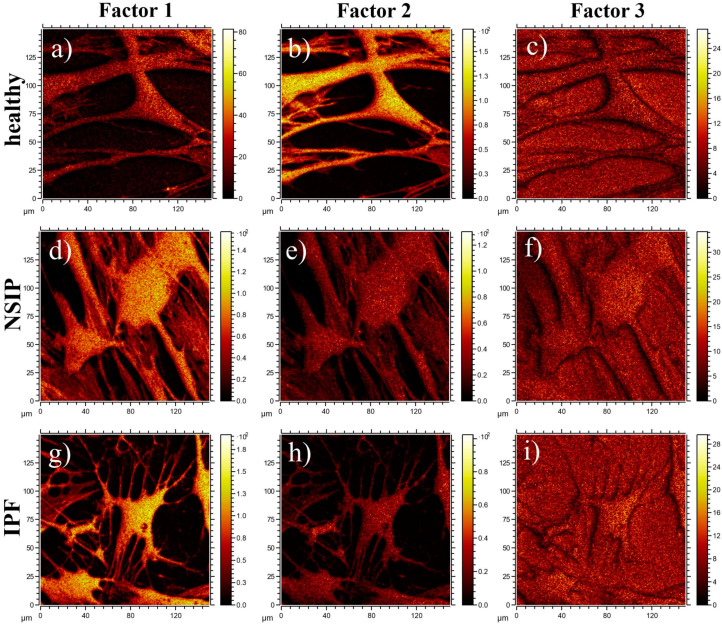
Spatial distribution of MCR factors 1 (**a**,**d**,**g**), 2 (**b**,**e**,**h**), and 3 (**c**,**f**,**i**) calculated for healthy (**a**–**c**), and NSIP- (**d**–**f**) and IPF-derived (**g**–**i**) fibroblasts.

The spatial distribution of ToF-SIMS signals corresponding to Factors 1, 2, and 3 calculated for all cell lines is presented in Figure 10. In all cases, images of Factor 3 are uniformly distributed and related to the silicon substrate. In turn, the distribution of Factors 1 and 2, corresponding to the loadings presented in Figure 9, are correlated to the position of cells, and enable discrimination between healthy and disordered fibroblasts, based on their chemical composition.

## 3. Discussion

### 3.1. Growth of Cells

The impact of substrate elasticity on the growth and viability of healthy, as well as IPF- and NSIP-derived fibroblasts was traced using fluorescence microscopy and MTS colorimetric assays, respectively.

For healthy cell lines (Figure 1) no significant differences in the number of cells was noticeable between the micrographs recorded for cells cultivated with PDMS A, PDMS D and glass. For each substrate, the monotonic growth of the number of cells was observed. After 24 h of culture, the cells formed a rare net with very limited cell-cell contacts. For longer culture times (72 h), the number of cells increased, and they started to form a monolayer, which became confluent after 144 h of culture. However, for the stiff substrate (PDMS D), the amount of cells initially observed was slightly lower compared to other substrates, which may be caused by the weaker adhesion of fibroblasts to this material, compared to the softer substrate [73]. In fact, the adhesion of fibroblasts to PDMS D was so weak that they easily detached, even during standard staining procedures (see Figure S1, Supplementary Materials). The preferential adhesion and proliferation of cells cultured on soft PDMS, compared to its stiff counterpart, have also been demonstrated for cancerous cells [74,75], where this effect was strong enough to impose a precise positioning of cells, driven by the elasticity pattern on the substrate [76].

A slightly adverse effect of PDMS D substrates on the growth of cells may also be concluded from their morphology—the cells seemed to have greater tension and thicker stress fibers, compared to cells cultured on other substrates. This result was in accordance with the literature data [47], showing that matrix elasticity within a pathophysiological range controls the contractile and proliferative functions of disordered and healthy lung fibroblasts, and may even lead to the transformation from fibroblasts into myofibroblasts [45,77]. For longer incubation times, the number of cells became similar for all substrates, suggesting an enhanced proliferation rate on the stiff substrate.

Analysis of the micrographs recorded for NSIP-derived fibroblasts (Figure 2) led to similar conclusions regarding the number of cells, which grew monotonically with increasing culture time, forming confluent monolayer after 144 h. However, for this cell line, the observed growth was slower compared to healthy cells. This effect was visible mainly for culture times equal to 72 h, where the number of NSIP-derived fibroblasts was reduced in comparison to their healthy counterparts. Moreover, the morphology of NSIP-derived cells differed significantly from that observed for healthy fibroblasts; NSIP-derived cells were flattened, and their spreading area was much greater than for healthy cells. Moreover, NSIP-derived fibroblasts also demonstrated features characteristic of a myofibroblastic phenotype, a hallmark of fibrosis [45,78], such as a large number of thick stress fibers, running parallel with the long axis of cell.

In the case of the last examined cell line, i.e., IPF-derived fibroblasts, the number of cells grew with increasing culture time, with no noticeable differences between different substrate materials, similar to the results for healthy and NSIP-derived fibroblasts. However, the number of cells was significantly reduced and cells did not form a monolayer even during the longest cultivation time (144 h). This effect was mainly visible for PDMS A and D, suggesting a weaker adhesion and slower proliferation of cells on PDMS-based substrates. It should be noted here that PDMS surface is highly hydrophobic [79], thus the ability of cells to adhere to this substrate is usually reduced. To overcome this problem, the PDMS substrates are usually modified, e.g., by coating with ECM proteins [80,81,82,83]. However, the spreading of cells on unmodified PDMS is possible for some cells [75,84], most likely due to the presence of serum proteins in the culture medium, serving as an adhesion matrix.

The recorded fluorescence micrographs also demonstrated that cell morphology changes with incubation time. Initially (24 h), cells cultivated on PDMS substrates had much greater tension compared to the control sample, but, for longer incubation times they become more flattened, with a large spreading area and thick stress fibers clearly visible. This rearrangement may be linked with the fact that, for longer times, cells usually develop an extracellular matrix, thus increasing their ability to adhere [85]. However, in our case, the observed change in cell shape, from elongated (characteristic for fibroblasts) into more polygonal form, as well as an increase in cell area, may be instead associated with the phenotypic transformation from fibroblasts into myofibroblasts [86], induced by an increase in the substrate elasticity [44,45,77]. Such a hypothesis is supported by the comparison between the fluorescence micrographs recorded for healthy (Figure 1) and disordered (Figure 2 and Figure 3) fibroblasts—for the latter cells, the spreading area is much greater than for healthy cells and their shape is far from elongated.

Based on the fluorescence micrographs, a strong impact of substrate elasticity on the growth of cells may be concluded. This hypothesis is supported by the quantitative analysis of the results, by means of the proliferation index and substrate cytotoxicity towards fibroblasts.

The analysis of the proliferation index (Figure 4) is in agreement with the observations made from fluorescence micrographs. For all cell lines, the proliferation index increased monotonically with increasing incubation time. However, the exact values depended strongly on the cell line and the substrate. For soft PDMS (Figure 4a), the proliferation index was comparable for healthy and NSIP-derived fibroblasts, and slightly but evidently lower for IPF-derived cells. In turn, for the two other substrates, i.e., PDMS D and the control sample, the calculated indices were highest for healthy cells, lower for NSIP-derived fibroblasts and the lowest for IPF-derived cells. This implies the most effective proliferation process for healthy cells, especially on stiff PDMS, which is intuitive, as adherent cells need to slightly detach from the surface to be able to divide, and detachment is favored on the PDMS D with very weak adhesive properties. In turn, proliferation was limited for both disordered cell lines. However, this effect was much stronger for fibroblasts derived from IPF, which is a significantly more severe disease compared to NSIP and may result in amplified alterations of the physic-chemical properties of cells. The presented results also indicate that healthy fibroblasts are the most sensitive to substrate elasticity. The comparison of the proliferation indices enables discrimination between healthy and IPF- and NSIP-derived cells for all substrates. Moreover, it enables discrimination between both disordered cells, for proliferation indices calculated on both PDMS-based substrates.

In turn, substrate cytotoxicity (Figure 5) varies the most for healthy fibroblasts cultured on substrates with different elasticity. For PDMS A, the number of these cells decreased below 80% compared to control sample, therefore, according to the ISO 10993-5 standard, this substrate may be considered as cytotoxic to healthy fibroblasts [87]. In turn, the cytotoxicity calculated for cells cultured for 24 h on PDMS D decreases to 50%. However, this effect may be associated with the reduced initial adhesion of healthy fibroblasts, postulated already for PDMS D, rather than with substrate cytotoxicity, as the number of cells for longer culture times is similar to that of the control substrate. The presented results also suggest that healthy fibroblasts are the most sensitive to the substrate elasticity and that the substrate cytotoxicity parameter may be used to identify this cell line. However, for the longest incubation time (144 h, Figure 5c), disordered fibroblasts cultured on PDMS D could also be distinguished from each other.

In turn, the cell viability (Figure 6) calculated for healthy fibroblasts was significantly higher than for other cell lines on all analyzed substrates. However, their growth was slightly slower on soft PDMS (Figure 6a). In turn, the viability determined for NSIP-derived fibroblasts cultured on PDMS A was only slightly lower, and was significantly reduced on PDMS D and the control sample, whereas for IPF-derived cells, it remained very low for all substrates. In a similar manner to the proliferation index and substrate cytotoxicity, MTS results were analyzed in terms of the possibility of discrimination between the examined cell lines. Except for cultures on the control sample for a time equal to 72 h, the MTS values determined for all substrates enabled us to unambiguously distinguish the healthy fibroblasts. However, the difference between NSIP- and IPF-derived cell lines was visible only for cells cultured on PDMS A substrate.

The main goal of the performed research was to define the set of parameters which would enable discrimination between cell lines, especially those derived from NSIP and IPF. Moreover, keeping in mind the potential diagnostic applications of the proposed parameters, they should be accessible after the shortest possible time, to increase the speed of diagnosis and reduce the expenses related with cell culture. The presented results indicate that the proliferation index preferably fits the demanded criteria. For all analyzed substrates, healthy fibroblasts could be distinguished from the disordered cells. Moreover, the differences between proliferation indices calculated for fibroblasts cultured on both PDMS substrates enabled discrimination between all considered cell lines, providing their identification after only 72 h of culture. Two other parameters, i.e., substrate cytotoxicity and cell viability, also enable discrimination between these cells but under a much more confined range of experimental conditions.

### 3.2. Elasticity

The fibrotic process occurring in the progress of ILDs is related with the reorganization of cytoskeletal architecture and an abnormal abundance of ECM components, such as collagen, which may result in altered mechanical properties of disordered cells compared to the healthy cells. However, the most interesting question is whether the mechanical properties of cells are also different for the IPF- and NSIP-derived fibroblasts, and whether this hypothetical difference is big enough to be detected. To verify this issue, the elasticity of fibroblasts was measured using AFM-based force spectroscopy. Research on the importance of the mechanical properties of the substrate in the fibrogenesis process [43], as well as the phenotypic differentiation of fibroblasts into myofibroblasts [44,45,88], suggests there is an enhancement of pathological processes with increased substrate elasticity. Thus, the measurements were performed on substrates of differing stiffness (PDMS A—600 kPa, PDMS D—1.5 MPa, glass—72 GPa), which should enhance any postulated difference in the elasticity of cells. As cells are complex objects, with the nuclei and various organelles characterized by different mechanical properties, the measurements were performed for 3 indentation depths, equal to 200, 400, and 600 nm, and providing information from different cell areas [66,67,68].

The measured E value varied from ~11.5 to 24.4 kPa, which is close to the physiological range of the Young modulus of pulmonary tissue (~2–10 kPa) reported elsewhere [46,47,89,90], and depended strongly on the cell type and experimental conditions. For cells cultured on the control substrate, the stiffness recorded for healthy cells was reduced by ~15–20% and 20–25%, compared to IPF- and NSIP-derived fibroblasts, respectively. The observed trend in cell stiffness was in accordance with those reported before—healthy fibroblasts are softer than disordered cells [89,91,92], which may indicate an impairment of disordered fibroblasts induced by the fibrosis process. This result also fits with the fluorescence visualization of the actin cytoskeleton architecture, where large numbers of stress fibers and their characteristic arrangement into parallel fibers which modulate cellular stiffness [93], was observed for NSIP- and IPF-derived fibroblasts cultured on glass (Figure 2 and Figure 3). In contrast, for cells cultured on PDMS D, the determined Young moduli were slightly higher for healthy cells, as compared to their disordered counterparts. This effect may be associated with the high responsiveness of healthy fibroblasts to the pathological elasticity of PDMS D, which may significantly alter their mechanical properties [45,46,47,48]. In turn, for PDMS A, the recorded stiffness was similar for all examined cells, except the IPF- and NSIP-derived fibroblasts examined at an indentation depth of 600 nm. The measured stiffnesses reveal that for all substrates, the Young modulus decreased for deeper cell regions, which is in agreement with the literature data [66,67,68].

With regard to the possibility of discriminating between the disordered cell lines, based on the comparison of the E values recorded for different cell types, the data presented show that it is possible exclusively for measurements performed at 600 nm on the soft substrate, where the Young moduli are equal to 17.47 ± 0.84 and 13.82 ± 0.54 kPa, for NSIP and IPF-derived cells respectively (bolded in Table 1). The healthy and NSIP-derived fibroblasts cannot be discriminated between under these experimental conditions, but this is still a very promising result, indicating that although the alterations of cell elasticity are very similar for both analyzed disorders, they still can be distinguished based on their mechanical properties. However, to increase the reliability of identification, it would be beneficial if the observed differences were more general than limited to a single set of experimental conditions. Fortunately, careful analysis of the results provides another analytical approach, based on the analysis of the ratio between Young moduli calculated for cells cultured on different substrates (Figure 7). The ratios between E values calculated on PDMS A and glass (Figure 7a) were comparable for both disordered cell lines and slightly, but visibly, higher for healthy fibroblasts, enabling their discrimination from other cells. In turn, the ratios between E values calculated on PDMS D and glass (Figure 7b) were significantly higher for healthy cells, compared to NSIP- and IPF-derived fibroblasts. However, the difference between the two disordered cell lines was also significant, for all indentation depths, providing an explicit identification of each one from all three analyzed cell lines. In contrast, for the ratios between Young moduli calculated on PDMS D and PDMS A (Figure 7c), both disordered cell lines could be easily distinguished, but the healthy and IPF-derived fibroblasts may not be discriminated between. The analysis presented here implies that the culture of cells on PDMS D substrate is crucial for their proper and unambiguous identification.

### 3.3. Chemical Analysis

The biochemical properties of lung tissue are strongly affected by the proceeding fibrotic processes, which are mostly advanced for IPF-derived fibroblasts. Therefore, the chemical composition should differ between the examined cell lines. To verify this hypothesis, ToF-SIMS measurements were performed for samples prepared using two procedures described in the Section 4.6, and accompanied by MCR and PCA analyses which provide information about any subtle differences in chemical composition.

PCA of data collected from cells fixed following Procedure I clearly separated all three cell lines. PC1 distinguished between IPF-derived (negative scores for PC1 and positive scores for PC2) and healthy cells (positive scores for PC1 and PC2). In turn, the second PC identified the difference in NSIP-derived cells (negative scores for PC2). Since the data points collected for healthy fibroblasts were placed in the 1st quadrant, the signals that loaded PC1 and PC2 positively (loadings for PC1 > 0 and loadings for PC2 > 1SD_PC2_ conditions) were the most characteristic for these cells. Similar analysis under conditions where loadings for PC1 < 0 and loadings for PC2 > 1SD_PC2_ pointed to fragments characteristic of IPF-derived cells. Finally, PCA analysis indicated that masses with loadings for PC2 lower than −1SD_PC2_ contributed strongly to the separation of NSIP-derived cells from healthy and IPF-derived cells. Similarly to the cells fixed following the first procedure, the ToF-SIMS signals originating from fatty acids were the most characteristic of IPF-derived fibroblasts. Moreover, Ca presence is detected in the deeper regions of these cells, which is in accordance with the literature data, suggesting that the calcification process is most effective for IPF [59,60]. Additionally, the presence of fatty acids in IPF-derived cells was reasonable, as they frequently accompany cholesterol, the biosynthesis and metabolism of which is disturbed in IPF disorder [94].

The results of the multivariate curve resolution (MCR) analysis of ToF-SIMS cell images (Figure 10) also enabled discrimination between healthy and disordered fibroblasts based on their chemical composition. In addition, the ToF-SIMS images revealed that the chemical structure of the cell surface is uniform in the whole cell area, without any visible structures (with size greater than 3 μm). Moreover, the images of Factor 1 showed a noticeably higher intensity of corresponding chemical components for IPF-derived fibroblasts as compared to the NSIP-derived cells, thus enabling discrimination between them.

## 4. Materials and Methods

### 4.1. Cell Culture

Normal human lung fibroblasts (NHLF) were purchased from Lonza (catalog number CC-2512), IPF-derived fibroblasts (LL97A) were purchased from ATCC (catalog number ATCC-CCL-191), and NSIP-derived fibroblasts (MN) were obtained from the patient sample, using the protocol described in the Section 2.2. All cell lines were cultured in a fibroblast-dedicated culture system containing FBM^TM^ Basal Medium (Lonza, Basel, Switzerland, catalog number CC-3131), and FGM^TM^ SingleQuots^TM^ supplements (Lonza, catalog number CC-4126) in culture flasks, in a CO_2_ incubator providing 95% air/5% CO_2_ atmosphere. The control glass sample, as well as the soft and stiff PDMS substrates attached to glass coverslips, were placed into a Petri dish (35 mm in diameter). Then, they were sterilized for one hour under UVC light (germicidal lamp, λ = 254 nm), in a laminar flow chamber (Nu425, NuAire). After sterilization, a solution of cells (80,000 cells per mL of the culture medium) was placed over glass or the PDMS surface. Next, the Petri dish was incubated in the CO_2_ incubator for 1, 3 and 6 days. To prove the reproducibility of the results, the experiments were repeated at least three times for each cell line and time-point. For each experimental sequence, two or three identical samples were prepared and measured.

### 4.2. Bronchial Bronchoscopy and Primary Cells Isolation

Primary human fibroblasts were cultured from bronchial biopsies from a donor with NSIP. The patient was treated in the 2nd Department of Internal Medicine (Department of Pulmonology), Jagiellonian University Medical College in Kraków. The patient was a 74 year old female. The diagnosis of NSIP had been established after careful exclusion of underlying processes known to be associated with NSIP (e.g., connective tissue diseases, environmental factors, potential causative drugs) by a multidisciplinary team, according to international guidelines [8,34]. The biopsy specimens were obtained from the segmental bronchi by a specialist in the Bronchoscopy Unit in the 2nd Department of Internal Medicine, according to the guidelines of the American Thoracic Society [95]. Bronchoscopy was carried out using the bronchofiberoscope BF 1T180 (Olympus, Tokyo, Japan), with local anesthesia (2% lidocaine) and mild sedation (0.05–0.1 mg fentanyl iv +2.5–5 mg midazolam iv).

Primary human bronchial fibroblasts (MN) were established from the bronchial biopsies according to the protocol developed earlier, with some modifications [96]. Explants were washed with cold phosphate buffered saline (PBS, Corning, Corning, NY, USA) with antibiotics (penicillin/streptomycin cocktail) (Sigma-Aldrich, Darmstadt, Germany) and transferred to collagenase IV (Worthington, USA; 1 mg/mL in Hank’s Balanced Salt solution with Ca and Mg ions) diluted to a final concentration in fibroblast growth medium (FGM; Fibroblast Basal Medium containing supplements from the FGM-2 Bullet Kit; Lonza). After 5–6 h of incubation at 37 °C with intensive mixing several times, the digested samples were centrifuged for 5 min at 300 g. Next, the pellet of cells with some undigested tissue fragments was shaken intensively for up to 2 min with 0.05% trypsin with EDTA (Sigma-Aldrich, Darmstadt, Germany), the trypsin was inactivated with fresh FGM, and the solution was re-centrifuged (5 min, 300 g). Primary cultures of HBFs were established on Petri dishes in FGM for c.a. 2–3 weeks, with FGM replacement every 48–72 h. Cells were cultured in vitro under standard conditions (37 °C and 5% CO_2_) in FGM or Dulbecco’s Modified Eagle Medium (DMEM) with high (4500 mg/L) glucose, supplemented with 10% fetal bovine serum (FBS, Gibco), and antibiotics (penicillin/streptomycin cocktail) up to 80–85% confluence, and were used for experiments after the third passage. For long-term storage cells were banking in FGM medium supplemented with 50% FBS and 10–15% sterile dimethyl sulfoxide (DMSO, Sigma-Aldrich, Darmstadt, Germany).

### 4.3. Preparation of PDMS Substrates

The PDMS mixture was prepared using commercially available Sylgard 184 (Dow Corning) with a base to curing agent mass ratio of 10:1. Next, it was admixed with benzophenone (Sigma-Aldrich, Darmstadt, Germany) at a mass ratio of 1:100 benzophenone to PDMS dissolved in xylene (200 mg/mL, POCH Gliwice, Grewicz, Poland), and degassed. The PDMS substrates were prepared on a 25 mm round coverslip glass using a spin-coating technique (KW-4A, Chemat Technology, Los Angeles, CA, USA), resulting in an elastomeric film with a thickness of ~60 µm. The spinning speed was set to 500 rpm. In order to obtain soft substrates, a fraction of substrates was exposed to UV light (400 W mercury lamp, providing uniform surface irradiation with a wavelength of 254 nm) for at least 5 h. The irradiated and non-irradiated (stiff, E = 1.5 MPa) samples were baked for 15 min at 150 °C, resulting in soft (E = 600 kPa) and stiff (E = 1.5 MPa) substrates.

### 4.4. Ethics Statement

This study was approved by the institutional review board, the Bioethics Committee of the Jagiellonian University (No 1072.6120.105.2019).

### 4.5. Force Spectroscopy

Force spectroscopy measurements and elasticity maps from cells were collected by using a commercially available atomic force microscope (model XE-120, Park System). The PDMS samples covered with a monolayer of cells were placed inside the Petri dish lid and filled with cell culture medium (FGM–2 Fibroblast Growth Medium-2 BulletKit, Lonza, catalog number: CC-3132). Samples prepared in this way were mounted on the AFM. The ORC8-10 D AFM probe was used, a commercially available silicon nitride from Bruker. The tip was immersed in medium and approached close to the cell’s surface. Force curves (i.e., vertical deflection vs. scanner position) were collected within a grid of 5 × 5 points from approximately 40 different cells, for each substrate and time point. To estimate the quantity of the relative Young’s modulus, only the approach section of the collected force curve was analyzed. It was recalculated into a force versus indentation curve. After that, the Hertz contact model was fitted, with a paraboloid approximation of the shape of the probing tip [97].

### 4.6. ToF-SIMS

For ToF-SIMS sample preparation, cells were cultured for 72 h on silicon wafers (1 × 1 cm^2^) using the same methodology as for PDMS and glass substrates (Section 2.1). Then, two procedures were used to fix cells to the substrate. In Procedure I, the first step was to pre-fix cells by adding a 1 mL solution of 3.7% of paraformaldehyde (Fluka) to the culture medium for 2 min at 37 °C. Next, cells were washed with phosphate-buffered saline (PBS, Sigma Aldrich) 3 times for 2 min. Then, to fix cells permanently, the samples were immersed in the same solution of 3.7% of paraformaldehyde (Fluka) for 15 min at room temperature, rinsed twice with PBS for 2 min, and dried in a nitrogen stream. In turn, in Procedure II, a drying protocol was applied using multistep washing of cells in diluted solutions of anhydrous alcohol [70].

**ToF-SIMS analysis** of normal and IPF-derived fibroblasts was carried out using the ToF-SIMS V instrument (ION.TOF GmbH, Munster, Germany) equipped with a 30 keV Bi_3_^+^ primary ion gun. Before analysis, the region of 400 µm × 400 µm was subjected to a gentle sputter with a 20 keV C60^+^ gun (1 frame at 1.2 nA) to remove any leftovers of the buffers and reagents used to fix the cells. Next, positive spectra were acquired from a 150 um × 150 um area located in the center of the sputter crater, with a total dose lower than 1 × 1012 ions/cm^2^.

Positive ion images were collected by summing the signal from 10 sequential analysis cycles, each consisting of 3 frames of 150 µm × 150 µm raster with Bi3^+^ at 0.08 pA for imaging, followed by a 1 frame of 400 µm × 400 µm area sputter a using C60^+^ at 1.2 nA. This summation protocol was used to acquire image data from a thicker layer of cells [98].

**ToF-SIMS data analysis**. Principal component analysis (PCA) and multivariate curve resolution (MCR) analysis were applied to analyze the collected spectra and images, respectively. All peak lists were generated manually and were composed with secondary ions of significant intensity. MCR analysis was performed using the ION.TOF SurfaceLab 7.2 software. PCA was carried out using the PLS Toolbox 7.5.2 (eigenvector Research, Manson, WA, USA) for MATLAB (MathWorks, Inc., Natick, MA, USA) software. Before PCA analysis, each recorded spectrum was normalized to the total counts and then, mean-centering was applied as a pre-processing method.

### 4.7. Colorimetric MTS Assay

The MTS colorimetric test (CellTiter 96 Aqueous One Solution Cell Proliferation Assay, Promega, Madison, WI, USA, catalog number: G3580) was used to verify the viability of living cells under the influence of a changing environment. Fibroblast cells were cultured in a 24-well plate in 1 mL of culture medium, on glass as a control and every type of PDMS base. After an appropriate time, 100 μL of the MTS reagent (tetrazolium compound) was added to all wells with cells in the culture medium. Subsequently, cells were incubated at 37 °C in 95% air/5% CO_2_ atmosphere, in the incubator (Nuaire, Plymouth, MN, USA) for 1 h. The whole final volume (1.1 mL) was carried to a 96-well plate-of 100 μL per well. The reduction in tetrazolium compound which is caused by viable cells, generates a formazan product which changes the color of the culture medium to purple. The level of color saturation depends on the number of living cells in each well. For each cell line (NHLF, LL97A, and MN) and time-point (after 24 h, 72 h, and 144 h of cell culture), the absorbance was measured at OD = 490 nm. Colorimetric assays were repeated at least three times and for the all-experimental run, two or three identical samples were prepared and tested alongside control wells, with and without cells.

### 4.8. Fluorescence Microscopy

For fluorescent staining of cell actin fibers and the cell nucleus, the following protocol was applied. Cells were cultured in a Petri dish (diameter: 35 mm) on a glass coverslip (diameter: 15 mm) coated with PDMS of the required elasticity. The first step was to pre-fix cells after the proper time-step by adding a 1 mL solution of 3.7% of paraformaldehyde (Fluka) to the culture medium for 2 min at 37 °C. Next, cells were washed with phosphate-buffered saline (PBS, Sigma Aldrich) 3 times for 2 min. Then, to fix cells permanently, the samples were immersed in the same solution of 3.7% of paraformaldehyde (Fluka) for 15 min at room temperature and after fixation, rinsed twice with PBS for 2 min. Afterwards, a solution of 0.2% Triton X-100 at 4 °C was added for 4 min, followed by washing the coverslips with PBS for 2 min. To dye the actin cytoskeleton, cells were incubated with a solution of Alexa Fluor 488 conjugated with phalloidin (Alexa Fluor 488 Phalloidin, Thermo Fisher Scientific, Waltham, MA, USA, catalog number: A12379) in dilution 1.5 U for 40 min, and then thoroughly washed 3 times for 2 min with PBS. Subsequently, the cells were incubated with a 1 µg/mL solution of Hoechst 34,580 dye (Thermo Fisher Scientific, catalog number: H21486) for 9 min to stain the cell nuclei, before washing 3 times for 2 min with PBS again. Finally, all of the liquid was removed from samples and cells on cover glass were embedded in a drop of resin (DePex, SERVA, catalog number: 18,243.01, SERVA). Glass slides were used to cover the cells. After that, all samples were placed in a darkened box to dry for 24 h. For each cell line (NHLF, LL97A, and MN) and time-point (after 24 h, 72 h, and 144 h of cell culture), the fluorescent images were collected using an Olympus IX51 microscope equipped with a 100 W Mercury light source (Olympus U-LH100HG), U-MWIG2 filter (λexit = 530–550 nm, λemit = 590 nm), and a U-MNB2 source (λexit = 470–490 nm, λemit = 520 nm). To study the growth of fibroblasts, the first filter was used to record images of actin filaments, while the latter was used to detect fluorescently-labeled cell nuclei. Fluorescent images were recorded using the XC30 digital camera (Olympus). The maximum resolution of images captured by this camera is 2080 × 1544 px. All images were recorded using CellSense Dimensions (Olympus) software with a 20× (Universal Plan Fluorite) lens. For each experimental run, 10 fluorescent images from two or three substrates with stained cells were collected.

### 4.9. Statistical Analysis

Statistical analysis was performed by multivariate ANOVA, using Origin (OriginLab, Northampton, MA, USA). Statistical significance between groups was determined by performing Bonferroni’s post hoc analysis. Statistical significance was achieved for *p* < 0.01 (*) or *p* < 0.05 (**).

## 5. Conclusions

In the presented paper, three types of fibroblasts, i.e., healthy cells as well as those derived from IPF and NSIP, were examined in order to find a set of physic-chemical parameters enabling unambiguous discrimination between the cell lines. To enhance any possible differences, all experiments were performed for cells cultured on soft and stiff PDMS substrates. The growth and morphology of cells were traced using fluorescence microscopy and analyzed quantitatively using two parameters, i.e., cell proliferation and substrate cytotoxicity indices. For all cell lines, the proliferation index increased monotonically with increasing incubation time. However, the exact values depended strongly on the cell line and the substrate, and imply the most effective proliferation process for healthy cells, which are also the most sensitive to the substrate elasticity. Limited proliferation is mostly visible for fibroblasts derived from IPF, which is a significantly more severe disease than NSIP. Moreover, a slightly adverse effect of PDMS D substrates on the growth of all examined cell lines was concluded based on their growth and morphology—cells seem to have greater tension and thicker stress fibers, compared to cells cultured on other substrates. This observation supports the hypothesis that matrix elasticity within a pathophysiological range controls the contractile and proliferative functions of disordered and healthy lung fibroblasts, and may even lead to the transformation of fibroblasts into myofibroblasts. Another analyzed issue, i.e., viability of cells, recorded using MTS assays was the highest for healthy fibroblasts on all analyzed substrates. In the case of disordered fibroblasts, the viability of IPF-derived cells remained very low for all substrates, whereas for NSIP-derived fibroblasts it depended on the substrate used. Considering the potential diagnostic applications of the presented parameters, healthy fibroblasts may be distinguished from disordered cells based on the proliferation index calculated for all analyzed substrates. Discrimination between cells stemming from the examined disorders is also possible, but only for precisely adjusted experimental conditions—IPF- and NSIP-derived fibroblasts may be distinguished by the proliferation index calculated for cells incubated for 72 h, and MTS results for cells cultured on soft PDMS. In turn, cell stiffness analyzed by the direct comparison of Young moduli, examined using AFM-based FS, showed strong dependence on the cell type and experimental conditions: for the control substrate, the healthy fibroblasts were softer than the disordered cells; for cells cultured on PDMS D, the determined Young moduli were slightly higher for healthy cells as compared to their disordered counterparts; and the recorded stiffness was similar for all examined cells cultured on PDMS A. These results enable discrimination between healthy and disordered cells for fibroblasts cultured on the control substrate, whereas differentiation between IPF- and NSIP-derived fibroblasts is possible only for measurements performed on soft PDMS with an indentation depth of 600 nm. However, the comparison of modifications of cell stiffness in response to the altered substrate elasticity, expressed as the ratio between the Young moduli determined for different substrates, provides unequivocal discrimination between all cell lines for E_PDMS D_/E_glass_. In addition to their physical properties, the chemical composition of the cells was determined using ToF-SIMS combined with sophisticated analytical tools, i.e., PCA and MCR. The recorded signals originating from fatty acids were found to be the most characteristic for IPF-derived fibroblasts, and Ca presence was detected for the deeper regions of these cells, which is in accordance with the literature data. Careful analysis of the obtained results enables discrimination between all examined cells, however, in this case the differences between IPF- and NSIP-derived fibroblasts were very subtle.

The research performed here shows that unambiguous discrimination between IPF- and NSIP-derived cells is possible, based on their different physical and chemical properties. However, this is a fine distinction and at least two of the proposed parameters should be examined independently to ensure the reliability of correct cell identification. In turn, discrimination based on the cellular response to the altered stiffness of the substrate provides an easy and direct tool enabling unequivocal identification of each cell line, including the discrimination between IPF- and NSIP-derived fibroblasts. Therefore, we believe that the mechanical properties of cells may serve as prospective diagnostic biomarkers, which should be considered when constructing novel tools dedicated for fast and reliable diagnosis of ILDs. An effective label-free identification of cells, enabling early-stage diagnosis and personalized therapy is the key issue, especially in case of IPF, where long-term survival is likely to increase as patients are diagnosed earlier and given treatments able to slow down disease progression.

## Figures and Tables

**Table 1:** Stiffness of healthy, NSIP- and IPF-derived fibroblasts (mean ± SE) determined for different substrates (PDMS A, PDMS D, glass) and for different indentation depths (200, 400, 600 nm).

	Young Modulus E [kPa]		
	Healthy	NSIP	IPF
PDMS A 200 nm	20.29 ± 0.76	20.86 ± 1.06	18.61 ± 0.67
PDMS A 400 nm	17.32 ± 0.91	17.93 ± 0.82	16.33 ± 0.60
PDMS A 600 nm	16.80 ± 0.82	**17.47 ± 0.84**	**13.82 ± 0.54**
PDMS D 200 nm	22.67 ± 0.83	19.27 ± 0.74	20.56 ± 0.59
PDMS D 400 nm	20.09 ± 0.79	16.40 ± 0.65	17.58 ± 0.56
PDMS D 600 nm	18.21 ± 0.92	15.37 ± 0.66	16.27 ± 0.50
control 200 nm	19.57 ± 0.89	24.36 ± 0.90	22.56 ± 0.83
control 400 nm	16.67 ± 0.75	21.47 ± 0.84	19.70 ± 0.81
control 600 nm	15.02 ± 0.71	20.22 ± 0.81	17.98 ± 0.63

**Table 2:** The most characteristic ToF-SIMS signals for each cell line (Procedure I), revealed by loadings plot analysis.

			NSIP			IPF		
Mass [u]	Determined Formula	Assignment	Mass [u]	Determined Formula	Assignment	Mass [u]	Determined Formula	Assignment
71.98	CNNa_2_^+^		28.02	CH_2_N^+^		29.04	C_2_H_5_^+^	fatty acid
87.98	CNONa_2_^+^		30.04	CH_4_N^+^	all amino acids	41.04	C_3_H_5_^+^	fatty acid
			104.99	C_4_H_2_O_2_Na^+^		43.06	C3H7^+^	fatty acid
			142.95	C_2_HPO_4_Na^+^		55.05	C_4_H_7_^+^	fatty acid
			164.94	Na_3_PO_4_H^+^		57.07	C_4_H_9_^+^	fatty acid
						58.07	C_3_H_8_N^+^	fatty acid
						67.06	C_5_H_7_^+^	fatty acid
						91.39	C_7_H_7_^+^	fatty acid

**Table 3:** The most characteristic ToF-SIMS signals for each cell line (Procedure II), revealed by loadings plot analysis.

Healthy			NSIP			IPF		
Mass [u]	Determined Formula	Assignment	Mass [u]	Determined Formula	Assignment	Mass [u]	Determined Formula	Assignment
28.02	CH_2_N^+^		30.04	CH_4_N^+^	all amino acids	27.02	C_2_H_3_^+^	fatty acid
38.96	K^+^		55.02	C_3_H_3_O^+^		39.02	C_3_H_3_^+^	
71.98	CNNa_2_^+^		56.05	C_3_H_6_N^+^	all amino acids	39.96	Ca^+^	
					except for Gly			
123.94	Na_2_PO_3_^+^		59.05	C_3_H_7_O^+^	all amino acids	43.05	C_3_H_7_^+^	fatty acid
					except for Gly			
			70.07	C_4_H_8_N^+^	multiple amino acids	56.96	CaOH^+^	
			84.05	C_4_H_6_NO^+^	multiple amino acids	81.96	CNOCa^+^	
			84.08	C_5_H_10_N^+^	multiple amino acids			

## Supplementary Materials

The following supporting information can be downloaded at: www.mdpi.com/article/10.3390/ijms23042162/s1.

## References

[1] Raghu G., Brown K.K. (2004). Interstitial lung disease: Clinical evaluation and keys to an accurate diagnosis. Clin. Chest Med..

[2] King T.E. (2005). Clinical advances in the diagnosis and therapy of the interstitial lung diseases. Am. J. Respir. Crit. Care Med..

[3] Moua T., Ryu J.H. (2019). Obstacles to early treatment of idiopathic pulmonary fibrosis: Current perspectives. Ther. Clin. Risk Manag..

[4] Sundar M.K. (2014). Sleep Apnea Management in “Possible IPF” and “Idiopathic NSIP”: A Case-Series. J. Sleep Disord. Ther..

[5] Vancheri C., Failla M., Crimi N., Raghu G. (2010). Idiopathic pulmonary fibrosis: A disease with similarities and links to cancer biology. Eur. Respir. J..

[6] Baratella E., Ruaro B., Giudici F., Wade B., Santagiuliana M., Salton F., Confalonieri P., Simbolo M., Scarpa A., Tollot S. (2021). Evaluation of correlations between genetic variants and high-resolution computed tomography patterns in idiopathic pulmonary fibrosis. Diagnostics.

[7] Yoon H.Y., Moon S.J., Song J.W. (2021). Lung Tissue Microbiome Is Associated with Clinical Outcomes of Idiopathic Pulmonary Fibrosis. Front. Med..

[8] Travis W.D., Costabel U., Hansell D.M., King T.E., Lynch D.A., Nicholson A.G., Ryerson C.J., Ryu J.H., Selman M., Wells A.U. (2013). An official American Thoracic Society/European Respiratory Society statement: Update of the international multidisciplinary classification of the idiopathic interstitial pneumonias. Am. J. Respir. Crit. Care Med..

[9] Du Bois R., King T.E. (2007). Challenges in pulmonary fibrosis 5: The NSIP/UIP debate. Thorax.

[10] Kim D.S., Nagai S. (2007). Idiopathic nonspecific interstitial pneumonia: An unrecognized autoimmune disease?. Am. J. Respir. Crit. Care Med..

[11] Glaspole I., Goh N.S.L. (2010). Differentiating between IPF and NSIP. Chron. Respir. Dis..

[12] Flaherty K.R., Andrei A.C., King T.E., Raghu G., Colby T.V., Wells A., Bassily N., Brown K., Du Bois R., Flint A. (2007). Idiopathic interstitial pneumonia: Do community and academic physicians agree on diagnosis?. Am. J. Respir. Crit. Care Med..

[13] Schär B. (2013). A brief overview of IPF and NSIP. Contin. Med. Educ..

[14] Salonen J., Purokivi M., Bloigu R., Kaarteenaho R. (2020). Prognosis and causes of death of patients with acute exacerbation of fibrosing interstitial lung diseases. BMJ Open Respir. Res..

[15] Ley B., Collard H.R., King T.E. (2011). Clinical course and prediction of survival in idiopathic pulmonary fibrosis. Am. J. Respir. Crit. Care Med..

[16] Souza C.A., Müller N.L., Flint J., Wright J.L., Churg A. (2005). Idiopathic pulmonary fibrosis: Spectrum of high-resolution CT findings. Am. J. Roentgenol..

[17] Fernández Pérez E.R., Daniels C.E., Schroeder D.R., St. Sauver J., Hartman T.E., Bartholmai B.J., Yi E.S., Ryu J.H. (2010). Incidence, prevalence, and clinical course of idiopathic pulmonary fibrosis a population-based study. Chest.

[18] Sauleda J., Núñez B., Sala E., Soriano J. (2018). Idiopathic Pulmonary Fibrosis: Epidemiology, Natural History, Phenotypes. Med. Sci..

[19] Agabiti N., Porretta M.A., Bauleo L., Coppola A., Sergiacomi G., Fusco A., Cavalli F., Zappa M.C., Vignarola R., Carlone S. (2014). Idiopathic Pulmonary Fibrosis (IPF) incidence and prevalence in Italy. Sarcoidosis Vasc. Diffus. Lung Dis..

[20] Ebner L., Christodoulidis S., Stathopoulou T., Geiser T., Stalder O., Limacher A., Heverhagen J.T., Mougiakakou S.G., Christe A. (2020). Meta-analysis of the radiological and clinical features of Usual Interstitial Pneumonia (UIP) and Nonspecific Interstitial Pneumonia (NSIP). PLoS ONE.

[21] Belloli E.A., Beckford R., Hadley R., Flaherty K.R. (2016). Idiopathic non-specific interstitial pneumonia. Respirology.

[22] Travis W.D., Hunninghake G., King T.E., Lynch D.A., Colby T.V., Galvin J.R., Brown K.K., Man P.C., Cordier J.F., Du Bois R.M. (2008). Idiopathic nonspecific interstitial pneumonia: Report of an American Thoracic Society Project. Am. J. Respir. Crit. Care Med..

[23] Tsuchiya Y., Takayanagi N., Sugiura H., Miyahara Y., Tokunaga D., Kawabata Y., Sugita Y. (2011). Lung diseases directly associated with rheumatoid arthritis and their relationship to outcome. Eur. Respir. J..

[24] Torrisi S.E., Pavone M., Vancheri A., Vancheri C. (2017). When to start and when to stop antifibrotic therapies. Eur. Respir. Rev..

[25] Maher T.M., Strek M.E. (2019). Antifibrotic therapy for idiopathic pulmonary fibrosis. Respir. Res..

[26] Sathiyamoorthy G., Sehgal S., Ashton R. (2017). Pirfenidone and Nintedanib for Treatment of Idiopathic Pulmonary Fibrosis. South Med. J..

[27] Belhassen M., Dalon F., Nolin M., Van Ganse E. (2021). Comparative outcomes in patients receiving pirfenidone or nintedanib for idiopathic pulmonary fibrosis. Respir. Res..

[28] Flaherty K.R., Fell C.D., Huggins J.T., Nunes H., Sussman R., Valenzuela C., Petzinger U., Stauffer J.L., Gilberg F., Bengus M. (2018). Safety of nintedanib added to pirfenidone treatment for idiopathic pulmonary fibrosis. Eur. Respir. J..

[29] Raghu G., Remy-Jardin M., Myers J.L., Richeldi L., Ryerson C.J., Lederer D.J., Behr J., Cottin V., Danoff S.K., Morell F. (2018). Diagnosis of idiopathic pulmonary fibrosis An Official ATS/ERS/JRS/ALAT Clinical practice guideline. Am. J. Respir. Crit. Care Med..

[30] Aburto M., Herráez I., Iturbe D., Jiménez-Romero A. (2018). Diagnosis of Idiopathic Pulmonary Fibrosis: Differential Diagnosis. Med. Sci..

[31] Furini F., Carnevale A., Casoni G.L., Guerrini G., Cavagna L., Govoni M., Sciré C.A. (2019). The role of the multidisciplinary evaluation of interstitial lung diseases: Systematic literature review of the current evidence and future perspectives. Front. Med..

[32] Cavagna L., Monti S., Grosso V., Boffini N., Scorletti E., Crepaldi G., Caporali R. (2013). The multifaceted aspects of interstitial lung disease in rheumatoid arthritis. Biomed. Res. Int..

[33] Yoo H., Hino T., Han J., Franks T.J., Im Y., Hatabu H., Chung M.P., Lee K.S. (2021). Connective tissue disease-related interstitial lung disease (CTD-ILD) and interstitial lung abnormality (ILA): Evolving concept of CT findings, pathology and management. Eur. J. Radiol. Open.

[34] Raghu G., Collard H.R., Egan J.J., Martinez F.J., Behr J., Brown K.K., Colby T.V., Cordier J.F., Flaherty K.R., Lasky J.A. (2011). An Official ATS/ERS/JRS/ALAT Statement: Idiopathic pulmonary fibrosis: Evidence-based guidelines for diagnosis and management. Am. J. Respir. Crit. Care Med..

[35] Flaherty K.R., King T.E., Raghu G., Lynch J.P., Colby T.V., Travis W.D., Gross B.H., Kazerooni E.A., Toews G.B., Long Q. (2004). Idiopathic interstitial pneumonia: What is the effect of a multidisciplinary approach to diagnosis?. Am. J. Respir. Crit. Care Med..

[36] Ruaro B., Baratella E., Confalonieri P., Wade B., Marrocchio C., Geri P., Busca A., Pozzan R., Andrisano A.G., Cova M.A. (2021). High-resolution computed tomography: Lights and shadows in improving care for SSc-ILD patients. Diagnostics.

[37] Peng M., Wang W., Qin L., Liu H., Qin M., Zheng W., Shi J., Xu W., Zhu Y. (2017). Association between nonspecific interstitial pneumonia and presence of CD20+ B lymphocytes within pulmonary lymphoid follicles. Sci. Rep..

[38] Salvatore M., Ishikawa G., Padilla M. (2018). Is it idiopathic pulmonary fibrosis or not?. J. Am. Board Fam. Med..

[39] Blokland K.E.C., Waters D.W., Schuliga M., Read J., Pouwels S.D., Grainge C.L., Jaffar J., Westall G., Mutsaers S.E., Prêle C.M. (2020). Senescence of ipf lung fibroblasts disrupt alveolar epithelial cell proliferation and promote migration in wound healing. Pharmaceutics.

[40] Azadeh N., Limper A.H., Carmona E.M., Ryu J.H. (2017). The Role of Infection in Interstitial Lung Diseases: A Review. Chest.

[41] Ramos C., Montaño M., García-Alvarez J., Ruiz V., Uhal B.D., Selman M., Pardo A. (2001). Fibroblasts from idiopathic pulmonary fibrosis and normal lungs differ in growth rate, apoptosis, and tissue inhibitor of metalloproteinases expression. Am. J. Respir. Cell Mol. Biol..

[42] Prasad S., Hogaboam C.M., Jarai G. (2014). Deficient repair response of IPF fibroblasts in a co-culture model of epithelial injury and repair. Fibrogenes. Tissue Repair.

[43] Vicens-Zygmunt V., Estany S., Colom A., Montes-Worboys A., Machahua C., Sanabria A.J., Llatjos R., Escobar I., Manresa F., Dorca J. (2015). Fibroblast viability and phenotypic changes within glycated stiffened three-dimensional collagen matrices. Respir. Res..

[44] Booth A.J., Hadley R., Cornett A.M., Dreffs A.A., Matthes S.A., Tsui J.L., Weiss K., Horowitz J.C., Fiore V.F., Barker T.H. (2012). Acellular normal and fibrotic human lung matrices as a culture system for in vitro investigation. Am. J. Respir. Crit. Care Med..

[45] Balestrini J.L., Chaudhry S., Sarrazy V., Koehler A., Hinz B. (2012). The mechanical memory of lung myofibroblasts. Integr. Biol..

[46] Ebihara T., Venkatesan N., Tanaka R., Ludwig M.S. (2000). Changes in extracellular matrix and tissue viscoelasticity in bleomycin-induced lung fibrosis: Temporal aspects. Am. J. Respir. Crit. Care Med..

[47] Marinković A., Liu F., Tschumperlin D.J. (2013). Matrices of physiologic stiffness potently inactivate idiopathic pulmonary fibrosis fibroblasts. Am. J. Respir. Cell Mol. Biol..

[48] Solon J., Levental I., Sengupta K., Georges P.C., Janmey P.A. (2007). Fibroblast adaptation and stiffness matching to soft elastic substrates. Biophys. J..

[49] Tsai M.J., Chang W.A., Liao S.H., Chang K.F., Sheu C.C., Kuo P.L. (2019). The effects of epigallocatechin gallate (EGCG) on pulmonary fibroblasts of idiopathic pulmonary fibrosis (Ipf)—a next-generation sequencing and bioinformatic approach. Int. J. Mol. Sci..

[50] Germanguz I., Aranda E., Xiong J., Kissel N., Nichols A., Gadee E., O’Neill J. (2019). Fibrotic human lung extracellular matrix as a disease-specific substrate for 3D in-vitro models of pulmonary fibrosis. J. Respir. Med. Lung Dis..

[51] Parra E.R., Kairalla R.A., De Carvalho C.R.R., Capelozzi V.L. (2007). Abnormal deposition of collagen/elastic vascular fibres and prognostic significance in idiopathic interstitial pneumonias. Thorax.

[52] Jaffar J., Yang S.H., Kim S.Y., Kim H.W., Faiz A., Chrzanowski W., Burgess J.K. (2018). Greater cellular stiffness in fibroblasts from patients with idiopathic pulmonary fibrosis. Am. J. Physiol. Lung Cell. Mol. Physiol..

[53] Faffe D.S., Zin W.A. (2009). Lung parenchymal mechanics in health and disease. Physiol. Rev..

[54] Miki H., Mio T., Nagai S., Hoshino Y., Nagao T., Kitaichi M., Izumi T. (2000). Fibroblast Contractility. Am. J. Respir. Crit. Care Med..

[55] Griese M., Kirmeier H.G., Liebisch G., Rauch D., Stückler F., Schmitz G., Zarbock R. (2015). Surfactant lipidomics in healthy children and childhood interstitial lung disease. PLoS ONE.

[56] Spickett C.M., Pitt A.R. (2015). Oxidative lipidomics coming of age: Advances in analysis of oxidized phospholipids in physiology and pathology. Antioxid. Redox Signal..

[57] Zuo Y.Y., Veldhuizen R.A.W., Neumann A.W., Petersen N.O., Possmayer F. (2008). Current perspectives in pulmonary surfactant—Inhibition, enhancement and evaluation. Biochim. Biophys. Acta Biomembr..

[58] Bjermer L., Hallgren O., Zhou X.H., Tykesson E., Åhrman E., Maccarana M., Wildt M., Eriksson L., Hallgren O. (2017). Increased deposition of glycosaminoglycans and altered structure of heparan sulfate in idiopathic pulmonary fibrosis. Int. J. Biochem. Cell Biol..

[59] Egashira R., Jacob J., Kokosi M.A., Brun A.L., Rice A., Nicholson A.G., Wells A.U., Hansell D.M. (2017). Diffuse pulmonary ossification in fibrosing interstitial lung diseases: Prevalence and associations. Radiology.

[60] Chan E.D., Morales D.V., Welsh C.H., McDermott M.T., Schwarz M.I. (2002). Calcium deposition with or without bone formation in the lung. Am. J. Respir. Crit. Care Med..

[61] Scruggs A.M., Grabauskas G., Huang S.K. (2020). The role of KCNMB1 and BK channels in myofibroblast differentiation and pulmonary fibrosis. Am. J. Respir. Cell Mol. Biol..

[62] Huang S.K., Scruggs A.M., McEachin R.C., White E.S., Peters-Golden M. (2014). Lung fibroblasts from patients with idiopathic pulmonary fibrosis exhibit genome-wide differences in DNA methylation compared to fibroblasts from nonfibrotic lung. PLoS ONE.

[63] Roach K.M., Wulff H., Feghali-Bostwick C., Amrani Y., Bradding P. (2014). Increased constitutive αSMA and Smad2/3 expression in idiopathic pulmonary fibrosis myofibroblasts is K_Ca_3.1-dependent. Respir. Res..

[64] Lee J.U., Chang H.S., Jung C.A., Kim R.H., Park C.S., Park J.S. (2020). Upregulation of Potassium Voltage-Gated Channel Subfamily J Member 2 Levels in the Lungs of Patients with Idiopathic Pulmonary Fibrosis. Can. Respir. J..

[65] Raczkowska J., Rysz J., Budkowski A., Lekki J., Lekka M., Bernasik A., Kowalski K., Czuba P. (2003). Surface patterns in solvent-cast polymer blend films analyzed with an integral-geometry approach. Macromolecules.

[66] Pogoda K., Jaczewska J., Wiltowska-Zuber J., Klymenko O., Zuber K., Fornal M., Lekka M. (2012). Depth-sensing analysis of cytoskeleton organization based on AFM data. Eur. Biophys. J..

[67] Guz N., Dokukin M., Kalaparthi V., Sokolov I. (2014). If Cell Mechanics Can Be Described by Elastic Modulus: Study of Different Models and Probes Used in Indentation Experiments. Biophys. J..

[68] Ding Y., Wang J., Xu G.K., Wang G.F. (2018). Are elastic moduli of biological cells depth dependent or not? Another explanation using a contact mechanics model with surface tension. Soft Matter.

[69] Gostek J., Awsiuk K., Pabijan J., Rysz J., Budkowski A., Lekka M. (2015). Differentiation between Single Bladder Cancer Cells Using Principal Component Analysis of Time-of-Flight Secondary Ion Mass Spectrometry. Anal. Chem..

[70] Bobrowska J., Moffat J., Awsiuk K., Pabijan J., Rysz J., Budkowski A., Reading M., Lekka M. (2016). Comparing surface properties of melanoma cells using time of flight secondary ions mass spectrometry. Analyst.

[71] Graham D.J., Castner D.G. (2012). Multivariate analysis of ToF-SIMS data from multicomponent systems: The why, when, and how. Biointerphases.

[72] Aram P., Shen L., Pugh J.A., Vaidyanathan S., Kadirkamanathan V. (2015). An efficient TOF-SIMS image analysis with spatial correlation and alternating non-negativity-constrained least squares. Bioinformatics.

[73] Raczkowska J., Orzechowska B. (2020). Effect of tuned elasticity and chemical modification of substrate on fibrotic and healthy lung fibroblasts. Micron.

[74] Raczkowska J., Prauzner-Bechcicki S. (2018). Discrimination between HCV29 and T24 by controlled proliferation of cells co-cultured on substrates with different elasticity. J. Mech. Behav. Biomed. Mater..

[75] Prauzner-Bechcicki S., Raczkowska J., Madej E., Pabijan J., Lukes J., Sepitka J., Rysz J., Awsiuk K., Bernasik A., Budkowski A. (2015). PDMS substrate stiffness affects the morphology and growth profiles of cancerous prostate and melanoma cells. J. Mech. Behav. Biomed. Mater..

[76] Raczkowska J., Prauzner-Bechcicki S. (2016). Precise positioning of cancerous cells on PDMS substrates with gradients of elasticity. Biomed. Microdevices.

[77] Hinz B. (2012). Mechanical aspects of lung fibrosis: A spotlight on the myofibroblast. Proc. Am. Thorac. Soc..

[78] D’Urso M., Kurniawan N.A. (2020). Mechanical and Physical Regulation of Fibroblast–Myofibroblast Transition: From Cellular Mechanoresponse to Tissue Pathology. Front. Bioeng. Biotechnol..

[79] Raczkowska J., Prauzner-Bechcicki S., Lukes J., Sepitka J., Bernasik A., Awsiuk K., Paluszkiewicz C., Pabijan J., Lekka M., Budkowski A. (2016). Physico-chemical properties of PDMS surfaces suitable as substrates for cell cultures. Appl. Surf. Sci..

[80] Palchesko R.N., Zhang L., Sun Y., Feinberg A.W. (2012). Development of polydimethylsiloxane substrates with tunable elastic modulus to study cell mechanobiology in muscle and nerve. PLoS ONE.

[81] Sun K., Xie Y., Ye D., Zhao Y., Cui Y., Long F., Zhang W., Jiang X. (2012). Mussel-inspired anchoring for patterning cells using polydopamine. Langmuir.

[82] Lee J.N., Jiang X., Ryan D., Whitesides G.M. (2004). Compatibility of mammalian cells on surfaces of poly(dimethylsiloxane). Langmuir.

[83] Chou S.Y., Lin C.Y., Cassino T., Wan L., LeDuc P.R. (2020). Probing coordinated co-culture cancer related motility through differential micro-compartmentalized elastic substrates. Sci. Rep..

[84] Park J.Y., Yoo S.J., Lee E.J., Lee D.H., Kim J.Y., Lee S.H. (2010). Increased poly(dimethylsiloxane) stiffness improves viability and morphology of mouse fibroblast cells. Biochip J..

[85] Raychoudhury S., Raychoudhury K., Millette C. (2011). Biotechnological evaluation of extracellular matrix proteins expressed by cultured testicular cells. J. Biotech Res..

[86] Michalik M., Pierzchalska M., Legutko A., Ura M., Ostaszewska A., Soja J., Sanak M. (2009). Asthmatic bronchial fibroblasts demonstrate enhanced potential to differentiate into myofibroblasts in culture. Med. Sci. Monit..

[87] López-García J., Lehocký M., Humpolíček P., Sáha P. (2014). HaCaT Keratinocytes Response on Antimicrobial Atelocollagen Substrates: Extent of Cytotoxicity, Cell Viability and Proliferation. J. Funct. Biomater..

[88] Janmey P.A., Fletcher D.A., Reinhart-King C.A. (2020). Stiffness sensing by cells. Physiol. Rev..

[89] Gabasa M., Duch P., Jorba I., Giménez A., Lugo R., Pavelescu I., Rodríguez-Pascual F., Molina-Molina M., Xaubet A., Pereda J. (2017). Epithelial contribution to the profibrotic stiff microenvironment and myofibroblast population in lung fibrosis. Mol. Biol. Cell.

[90] Brown A.C., Fiore V.F., Sulchek T.A., Barker T.H. (2013). Physical and chemical microenvironmental cues orthogonally control the degree and duration of fibrosis-associated epithelial-to-mesenchymal transitions. J. Pathol..

[91] Sarna M., Wojcik K.A., Hermanowicz P., Wnuk D., Burda K., Sanak M., Czyö J., Michalik M. (2015). Undifferentiated bronchial fibroblasts derived from asthmatic patients display higher elastic modulus than their non-asthmatic counterparts. PLoS ONE.

[92] Liu F., Mih J.D., Shea B.S., Kho A.T., Sharif A.S., Tager A.M., Tschumperlin D.J. (2010). Feedback amplification of fibrosis through matrix stiffening and COX-2 suppression. J. Cell Biol..

[93] Gavara N., Chadwick R.S. (2016). Relationship between cell stiffness and stress fiber amount, assessed by simultaneous atomic force microscopy and live-cell fluorescence imaging. Biomech. Model. Mechanobiol..

[94] Gowdy K.M., Fessler M.B. (2013). Emerging roles for cholesterol and lipoproteins in lung disease. Pulm. Pharmacol. Ther..

[95] Sokolowski J.W., Burgher L.W., Jones F.L., Patterson J.R., Selecky P.A. (1987). Guidelines for fiberoptic bronchoscopy in adults. American Thoracic Society. Medical Section of the American Lung Association. Am. Rev. Respir. Dis..

[96] Wnuk D., Paw M., Ryczek K., Bochenek G., Sładek K., Madeja Z., Michalik M. (2020). Enhanced asthma-related fibroblast to myofibroblast transition is the result of profibrotic TGF-β/Smad2/3 pathway intensification and antifibrotic TGF-β/Smad1/5/(8)9 pathway impairment. Sci. Rep..

[97] Kuznetsova T.G., Starodubtseva M.N., Yegorenkov N.I., Chizhik S.A., Zhdanov R.I. (2007). Atomic force microscopy probing of cell elasticity. Micron.

[98] Graham D.J., Castner D.G. (2013). Image and Spectral Processing for ToF-SIMS Analysis of Biological Materials. Mass Spectrom..

