# Supplementary material for: Discrimination between NSIP- and IPF-Derived Fibroblasts Based on Multi-Parameter Characterization of Their Growth, Morphology and Physic-Chemical Properties
Source: Int J Mol Sci. 2022 Feb 15;23(4):2162. doi: 10.3390/ijms23042162 (PMC8880018; doi:10.3390/ijms23042162)
Supplement: Supplementary file 1 [file ijms-23-02162-s001.zip › ijms-1585368-supplementary.pdf]

Discrimination between NSIP- and IPF-derived fibroblasts based on multi-parameter characterization of their morphology, growth and physic-chemical properties.

Barbara Orzechowska[1], Kamil Awsiuk[2,3], Dawid Wnuk[4], Joanna Pabijan[1], Tomasz Stachura[5], Jerzy Soja[5], Krzysztof Sładek[5], Joanna Raczkowska[2,3]*

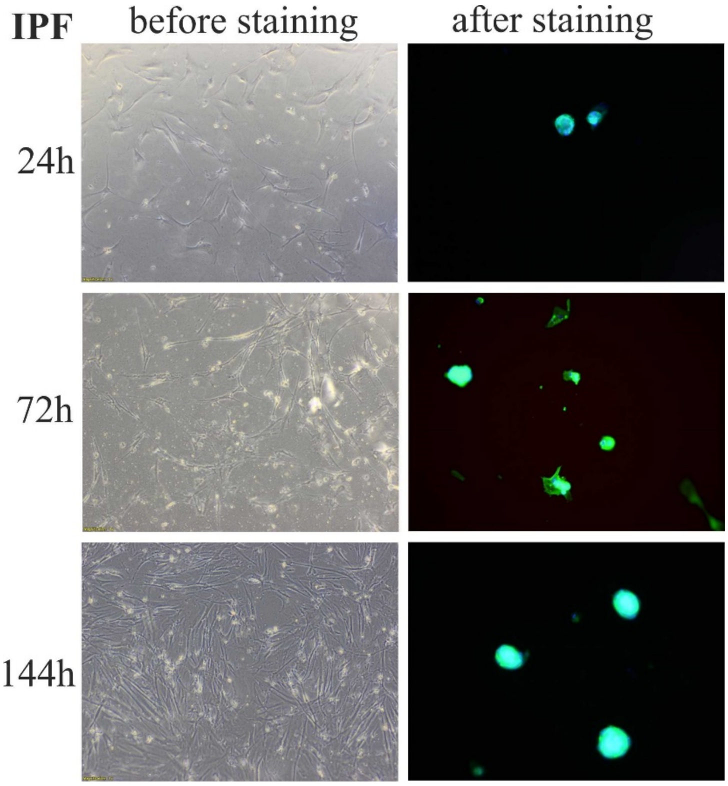

Figure S1. Fibroblasts cultured on PDMS D substrate. Optical image before staining (a) and fluorescence micrographs after staining (b), showing detachment of cells during staining procedure.
